# A Plasmid System That Utilises Phosphoribosylanthranilate Isomerase to Select Against Cells Expressing Truncated Proteins

**DOI:** 10.3390/biom15030412

**Published:** 2025-03-14

**Authors:** Aditi A. Ghuge, Susanne Gottfried, Anja H. Schiemann, Evelyn Sattlegger

**Affiliations:** 1 School of Food Technology and Natural Sciences, Massey University, Palmerston North 4410, New Zealand; a.ghuge@massey.ac.nz (A.A.G.);; 2 School of Natural Sciences, Massey University, Auckland 0632, New Zealand; 3 Maurice Wilkins Centre for Molecular Biodiscovery, Palmerston North 4442, New Zealand

**Keywords:** protein–protein interaction, nonsense mutation, truncated protein, degradation-prone protein, screening, mutagenesis

## Abstract

We have generated a vector that enables the removal of plasmids coding for truncated proteins. This vector expresses a protein of interest in the yeast *Saccharomyces cerevisiae* from a galactose-inducible promoter. The gene of interest is fused in-frame to a downstream sequence coding for phosphoribosylanthranilate isomerase (PRAI), which catalyses the third step in tryptophan biosynthesis. As a consequence, only the full-length protein of interest renders the host cell tryptophan prototrophic, allowing for selection against cells expressing truncated proteins. Our proof-of-principle study demonstrates that PRAI is functional when fused C-terminally to a protein, robustly rendering cells tryptophan prototrophic. The N-terminal GST tag and C-terminal myc tag allow for tag-mediated protein purification, co-precipitation studies, determination of relative expression levels, as well as validation of full-length expression of the protein via Western blotting.

## 1. Introduction

Mutagenesis approaches are essential for advancing our understanding of fundamental biological processes and are also crucial for various industrial applications [1,2]. This includes the dissection of protein function and studying the relationship between protein structure and function, such as identifying active sites, binding sites, and regions critical for protein stability. Other examples include simulating evolutionary processes in the laboratory, enhancing or altering the biological functions of proteins, modifying or engineering metabolic or signalling pathways (or creating new ones), and mapping these pathways to determine the sequence of events and the interdependence of various components.

Mutations in genes can lead to the expression of undesired truncated proteins. Several methods exist to identify such truncated proteins. This includes allele specific PCR and RFLP (restriction fragment length polymorphism [3]) analysis, but either method requires prior knowledge of the type of mutation. High-Resolution Melting Analysis cannot be conducted with large DNA fragments, and requires costly equipment [4]. Sequencing of the entire reading frame would detect any mutation; however, if many plasmids need to be analysed, this can be time-consuming and expensive. Western blotting can detect truncated proteins if the truncation is large enough to produce a detectable size difference. If the full-length protein contains a C-terminal tag, any truncation could be detected using an epitope-tag specific antibody. However, Western blotting is costly and time-consuming, particularly when a large number of protein variants need to be investigated. Nevertheless, removing truncated genes from a mutation library is crucial to allow efficient screening of mutated full-length proteins.

Here, we present a system designed for efficient removal of truncated genes without relying on biochemical, labour intensive, or costly methods. Notably, this system operates independently of the functionality of the protein of interest. To achieve this, we have developed a vector that facilitates the expression of the protein of interest in the yeast *Saccharomyces cerevisiae* from a galactose-inducible promoter. *S. cerevisiae* was chosen as the host organism because it is a well-established eukaryotic model organism that is easy and cheap to maintain and manipulate and has many favourable features, such as its capacity to express eukaryotic genes and to allow in-yeast recombination for the assembly of plasmids [5,6,7,8]. Furthermore, *S. cerevisiae* has high transformation efficiency, yielding more than 1 × 10^5^ transformants per microgram of vector DNA, where practically all transformants are true transformants carrying the transformed plasmid [8,9].

The gene of interest is fused in-frame to a C-terminal sequence coding for phosphoribosylanthranilate isomerase (PRAI), which catalyses the third step in the tryptophan (Trp) biosynthesis pathway [10]. As a consequence, only the expression of full-length protein restores tryptophan prototrophy, enabling the selective growth of cells expressing the full-length protein while eliminating those expressing truncated proteins. We conducted a proof-of-principle study to test the vector, using the RWDBD fragment of the protein General control non-derepressible 1 (Gcn1) as protein of interest. We found that the PRAI was functional when fused C-terminally to the RWDBD, rendering the host cell Trp prototrophic. The C-terminal addition of PRAI did not affect the functional property of the RWDBD, which is inhibition of the protein kinase Gcn2. Furthermore, the N-terminal GST tag and C-terminal myc tag were detectable in Western blots. Thus, our findings demonstrate that the Trp prototrophy conferred by the C-terminal PRAI domain allows for the counterselection of host cells harbouring plasmids that code for truncated genes.

## 2. Materials and Methods

### 2.1. Yeast Strains and Plasmids Used

The yeast strains and plasmids used in this study are outlined in Table 1 and Table 2. Plasmids were generated commercially (Genscript, Piscataway, NJ, USA) by inserting chemically synthesised DNA fragments into the provided vector. The sequences of the vectors generated in this work are provided as Supplementary Materials. Yeast synthetic media containing glucose (2%) or galactose (2%), and yeast peptone dextrose medium, with or without 2% agar, were prepared as published previously [8].

**Table 1:** Strains used in this study.

Strain	Genotype	Source
**Genetic Background H1511**		
H1511	*MATα ura3-52 trp1-63 leu2-3,112, GAL2^+^*	[11]
H2556	same as H1511 but *gcn1Δ*	[12]

**Table 2:** Plasmids used in this study.

Plasmid	Gene	Selectable Marker	Vector	Source
**Yeast Plasmids Containing a Galactose-Inducible Promoter**				
pES128-9	*GST*	*Amp^R^*, *URA3*, *leu2Δ*	vector, 2µ	[12]
pEG(KT)	*GST*	*Amp^R^*, *URA3*, *Leu2d*	vector, 2µ	[13]
pES124-B2	*GST-RWDBD*	*Amp^R^, URA3, Leu2d*	pES128-9, 2µ	[12]
pES167-2E	*GST-RWDBD^R2259A^*	*Amp^R^*, *URA3*, *Leu2d*	pES128-9, 2µ	[12]
pAG40	*GST-PRAI-myc*	*Amp^R^*, *URA3*, *Leu2d*	pEG(KT), 2µ	This work
pAG41	*GST-PRAI-myc*	*Amp^R^*, *URA3*, *leu2Δ*	pES128-9, 2µ	This work
pAG01	*GST-RWDBD-PRAI-myc*	*Amp^R^*, *URA3*, *leu2Δ*	pAG41, 2µ	This work
pSG50	*GST-RWDBD^R2259A^-PRAI-myc*	*Amp^R^, URA3, Leu2d*	pAG40, 2µ	This work
**Yeast Vectors for Expressing Genes from Their Native Promoter**				
pRS316	*empty vector*	*Amp^R^*, *URA3*	Vector, 2µ	[14]
pRS314	*empty vector*	*Amp^R^*, *TRP1*	Vector, 2µ	[14]

#### Semi-Quantitative Growth Assay (SQGA)

Semi-quantitative growth assays were conducted according to our previously published work [6]. Briefly, 10-fold serial dilutions were generated from saturated cultures, and 5 µL of each dilution was transferred onto solid minimal medium containing as carbon source either glucose or galactose, supplements to cover auxotrophies, and additional drugs as indicated in the figures. Plates were incubated at 30 °C and cell growth was documented over 14 days using a document scanner.

### 2.2. Generation of Whole Cell Extracts

Cell extracts were generated as published previously [15]. Briefly, saturated yeast cultures were inoculated in liquid minimal medium and grown to the exponential phase at 30 °C and 150 rpm. At an OD_600_ of 0.6–0.8, cultures were transferred into a mix of ice chips and formaldehyde (1% final concentration). After incubation for 1 h, inverting every 15 min, excess formaldehyde was quenched by adding glycine (0.25 M final concentration). Cell pellets were washed with ice-cold double de-ionised water and stored at −80 °C. For chemical cell lysis, 1/10th of the cell pellet volume was resuspended in 200 µL of 0.1 M NaOH. After 5 min at room temperature, cells were pelleted at ~1500× *g* for 5 min and the pellet resuspended in 200 µL of 2× denaturing protein sample buffer [4% SDS, 20% glycerol, 120 mM Tris–HCl pH 6.8, bromophenol blue (0.1%); just before use, 10% (*v*/*v*) 2-mercaptoethanol was added], and then incubated for 8 min at 80 °C.

### 2.3. Western Blotting

Equal volumes of lysed cell pellets were subjected to Western blotting using SDS denaturing polyacrylamide gel electrophoresis (SDS-PAGE) and a gradient gel [15,16]. Proteins were transferred to a nitrocellulose membrane (Biorad, Hercules, CA, USA, #1620112) and incubated with rabbit polyclonal antibodies against GST (1:5000; #SC-459, Santa Cruz, Dallas, TX, USA) or mouse monoclonal antibodies against the myc tag (1:500, #SC-40, Santa Cruz) or Pgk1 (1:5000; #459250; Pierce, Life Technologies, Waltham, MA, USA). Immune complexes were detected using horseradish peroxidase conjugated to donkey anti-rabbit antibodies (1:100,000, #31458, Thermo Scientific) or goat anti-mouse antibodies (1:50:000, #31430, Thermo Scientific), the Pierce ECL Western Blotting Substrate (Thermo Scientific, Waltham, MA, USA), and the ChemiDocTM Image System (BioRad).

### 2.4. Competitive Growth Assay

Optimisation of the competitive growth assay requires two strains, the original control strain (expressing the original protein of interest, or alternatively a strain with a fitness similar to that of the strain expressing the original protein of interest) and a positive control strain that exhibits increased fitness due to the enhanced features of the desired variant of the protein of interest (the positive control strain). If the desired protein of interest is unavailable, an alternative strain with enhanced fitness—equivalent to that conferred by the desired protein variant—can be used.

The plasmid backbone used for the original control strain harboured the *URA3* selectable marker but lacked the *Leu2d* gene (vector pAG41), while the plasmid backbone used for the positive control strain (vector pAG40) harboured both the *URA3* and *Leu2d* selectable markers.

Saturated overnight cultures were prepared using liquid media in which these strains grow equally well. Then, for the competition assay, a 250 mL flask containing 50 mL of fresh liquid medium that gave the positive control strain a growth advantage over the original control strain was used. In our study, this was synthetic minimal medium containing leucine (Leu), uracil (Ura), galactose as a carbon source, and 0.5 mM 3AT. This medium was inoculated with a starting OD that would—after 24 h of growth—lead to an OD_600_ of about 1. In our study, the starting OD_600_ was 0.05. The inoculum consisted of 1 part of the positive control strain, and 9 parts of the original control strain. We used the wild-type strain expressing RWDBD and RWDBD^R2259A^ as the original and positive control strains, respectively. As a control, the same competitive growth assay was set up, but the medium did not contain any 3AT.

The cultures were grown at 30 °C and 180 rpm. After 22–24 h of growth, an aliquot of the culture was taken and re-inoculated in a new 250 mL flask containing fresh 50 mL medium with a starting OD that was similar to that of the OD used when starting the competitive growth assay. In our case, we transferred about 1 mL of the culture. Again, the flasks were incubated, aliquots taken, and the culture re-inoculated, as described above. This process was repeated until the positive control strain had become the predominant strain over the original control strain. The relative abundance of these strains was determined using the fresh aliquots taken above, using two approaches. One was the colony counting approach and the other was the liquid culture PCR-mediated approach, as described below.

For the colony counting assay, aliquots were 10-fold serially diluted, and 0.1 mL of serially diluted aliquots were spread onto solid medium containing glucose as carbon source, where one set of plates lacked Ura and the other lacked both Ura and Leu. Plates were incubated at 30 °C until colonies emerged. On plates lacking Ura, all strains were expected to grow, while on plates lacking both Ura and Leu, only the positive control strains that harboured the plasmid carrying the *URA3* and *Leu2d* selectable markers were expected to grow. The colonies on each plate were counted to determine the abundance ratio between the original control strain and the positive control strain in the culture. Results were plotted in a graph as % abundance.

In parallel, the above fresh aliquots were used for the liquid culture PCR procedure, as described in the next section.

## 3. Liquid Culture PCR and Restriction Digest

A 1 µL volume of fresh yeast culture aliquots obtained from the competitive growth assay was added to a final volume of 20 µL Kapa Polymerase PCR mixture. PCR amplifications were carried out according to the manufacturer’s protocol (Roche, Basel, Switzerland, #KK2502) using primers ES2963s (ATAGGCAAAACACAGTTAGGG, sense primer) and ES2964r (AAATTGACAATGTTTGTCTTAGAA, reverse primer).

Amplicons were resolved on a 1.5% agarose gel and visualised using ethidium bromide and the ChemiDocTM Image System (BioRad). For restriction digest of the PCR amplicon, 3 µL of the PCR sample was directly added to a final volume of 20 µL restriction digest mixture containing 1× buffer and 0.2 µL of *AseI* (2 Units, New England Biolabs, Ipswich, UK, R0526S). After overnight incubation at 37 °C, samples were resolved on a 1.8% agarose gel and bands detected as above.

## 4. Results and Discussion

### 4.1. Generation of the Vector System

**Figure 1:**
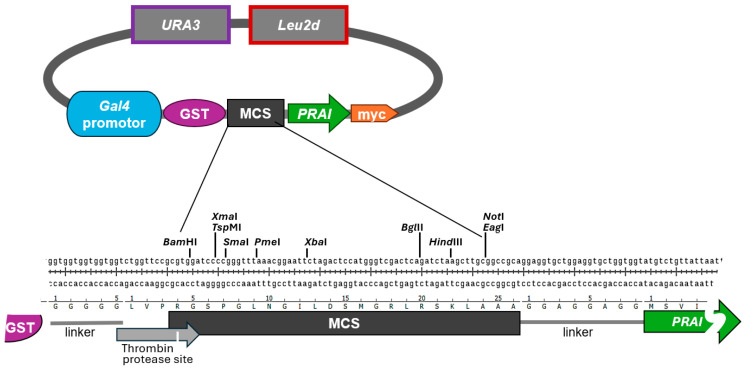
Cloning vectors that allow for galactose-induced expression of a protein of interest fused C-terminally to phosphoribosylanthranilate isomerase (PRAI), encoded by *TRP1* (for simplicity, the gene here is dubbed *PRAI)*, and decorated with the two epitope tags GST and myc. Shown is vector pAG40 containing the two selectable markers *URA3* and *Leu2d*. Vector pAG41 is identical but lacks the *Leu2d* marker. Below the plasmid map is shown the multiple cloning site (MCS) with its restriction sites, the linkers situated between the MCS and the N- and C-terminal tags, as well as the Thrombin protease site. The sequences of the vectors are provided as Supplementary Materials.

To select against strains expressing truncated versions of the protein of interest, we chose as the selectable marker the *TRP1* gene, coding for phosphoribosylanthranilate isomerase (PRAI). PRAI is an enzyme in the Trp biosynthetic pathway, conferring Trp prototrophy in *trp1Δ* strains [10]. We generated a vector system by modifying the vectors pEG(KT) and pES128-9 [12,13]. Our vectors contain a multiple cloning site (MCS) with a selection of common restriction sites flanked by sequences encompassing a galactose-inducible promoter and coding for an N-terminal GST tag followed by a thrombin protease site, and by downstream sequences encompassing the *TRP1* open reading frame followed by a myc tag (Figure 1). When grown on medium containing glucose as a carbon source, the galactose-inducible promoter is inhibited [13]. This allows for the maintenance of plasmid-bearing yeast cells without burdening them with the constitutive expression of potentially deleterious genes. When using galactose as the sole carbon source, the expression of genes under the galactose-inducible promoter is induced, while the additional presence of glucose suppresses the induction [17]. Raffinose does not inhibit the galactose-inducible promoter, allowing the easy induction of gene expression by adding galactose to liquid medium containing raffinose as a carbon source [18]. The resulting plasmids pAG41 (modified from vector pES128-9) and pAG40 (modified from vector pEG(KT) only differ in their selectable markers for plasmid maintenance. While pAG41/pES128-9 only contains *URA3* to confer Ura prototrophy, pAG40/pEG(KT) also contains *Leu2d* to confer Leu prototrophy.

Next, we wanted to confirm whether a protein of interest remains functional when expressed by our vector system, and whether the PRAI retains its enzymatic activity when fused to additional sequences at its N- and C-termini. We chose as the protein of interest a fragment of the protein General control non-derepressible 1 (Gcn1). Together with Gcn2, Gcn1 is part of a signal transduction pathway system best known for its role in enabling cells to cope with nutrient starvation [19,20]; in particular, starvation for amino acids. Direct interaction between Gcn2 and Gcn1 is essential for Gcn2 to sense amino acid shortage. Gcn2 is a protein kinase, and starvation stimulates Gcn2 and the Gcn2 signalling pathway. This ultimately leads to a shift in the cell’s gene expression profile, thereby allowing the cell to adapt and overcome starvation. Only when cells are able to overcome starvation can they continue to grow; cells that are unable to activate Gcn2 are unable to grow on starvation medium. A region in Gcn1 called the RWD binding domain (RWDBD) harbours the Gcn2 binding site. It has been documented previously that overexpressed RWDBD binds to Gcn2, thereby competing with endogenous Gcn1 for Gcn2 binding. This impairs endogenous Gcn1-Gcn2 interaction, consequently hampering Gcn2 activation and cell growth [12]. In our study, we chose the RWDBD region of Gcn1, with its biological function being the binding to Gcn2. The wild-type yeast strain used was H1511, which lacks the *TRP1* gene coding for PRAI [11].

### 4.2. PRAI Fused to Another Protein Still Confers Trp Prototrophy

**Figure 2:**
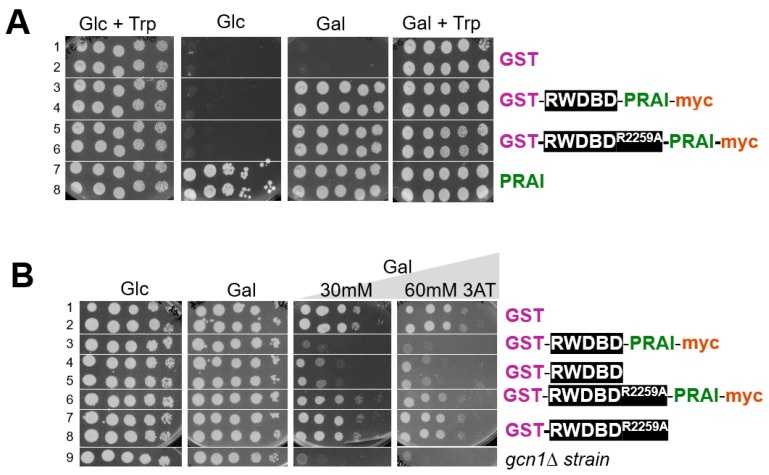
The proteins retain their function within the fusion construct. (**A**) PRAI still confers Trp prototrophy when C-terminally attached to the RWDBD of Gcn1. Wild-type yeast H1511 was transformed with plasmids expressing from a galactose-inducible promoter the recombinant proteins as indicated (lanes 1 and 2: plasmid pES128-9, lanes 3 and 4: pAG01, lanes 5 and 6: pSG50, lanes 7 and 8: pRS314 and pRS316). Independent transformants were grown to saturation, the culture subjected to 10-fold serial dilutions, and 5 µL of each dilution transferred to solid medium containing glucose (Glc) or galactose (Gal) as indicated, and Trp if indicated. (**B**) The RWDBD is still functional when decorated C-terminally with PRAI. Wild-type yeast H1511 was transformed with plasmids expressing from a galactose-inducible promoter the recombinant proteins as indicated (from top to bottom plasmids pES128-9, pAG01, pES124-B2, pSG50, pES167-2E, and pRS316). Independent transformants were subjected to semiquantitative growth assays as in (**A**), but all the plates contained Trp and 3AT, as indicated, to elicit starvation for His. As a control, we included the isogenic *gcn1Δ* strain H2556 transformed with a vector covering the Ura auxotrophy (vector pRS316).

We first wanted to test whether PRAI is still functional when fused to another protein, such as the RWDBD used in our study. If the PRAI retains its function when fused to another protein, then expression of this recombinant protein should render the strain Trp prototrophic. To test this, strain H1511 was transformed with plasmids expressing recombinant proteins consisting of only GST and RWDBD (GST-RWDBD), and consisting of GST, RWDBD, PRAI, and the myc tag (GST-RWDBD-PRAI-myc). The backbone of these plasmids has in common the *URA3* selectable marker, allowing plasmid maintenance in medium lacking Ura. To have a Ura and Trp prototrophic strain as a reference, H1511 was also transformed with two empty vectors bearing the *URA3* (pRS316) and *TRP1* (pRS314) selectable markers, respectively. Next, saturated overnight cultures were subjected to 10-fold serial dilutions and aliquots transferred to solid medium. Plates were incubated at 30 °C and the growth monitored with a document scanner. On medium containing glucose and Trp, all strains grew equally well, as expected (Figure 2A, left panel). On medium containing galactose and Trp, all strains were able to grow (Figure 2A, right panel). However, on medium containing galactose but not Trp, strains lacking the *TRP1* gene were unable to grow (Figure 2A, third panel, rows 1–2), validating that growth on this medium requires Trp prototrophy. On the same medium, strains expressing proteins fused to PRAI were able to grow, and they grew as well as the strain expressing plasmid-borne PRAI from its native promoter (Figure 2A, third panel, rows 3–6 vs. 7 and 8). This suggests that the PRAI fused to the RWDBD is still enzymatically functional. As expected, on medium containing glucose but not Trp, strains expressing native PRAI from its native promoter were able to grow, but not strains expressing the recombinant proteins harbouring PRAI from the galactose-inducible promoter (Figure 2A, second panel, rows 7 and 8 vs. 3–6).

Together, our findings strongly suggest that PRA1—when C-terminally myc-tagged and N-terminally fused to another protein—is still able to confer Trp prototrophy. We are aware of the fact that PRAI function may be impacted by the type of protein it is fused to. However, we refrained from testing proteins other than the RWDBD, as one cannot forecast which proteins may impact PRAI function, if at all. To reduce the likelihood of steric interference between the protein of interest and PRAI, the vector is constructed such that the protein of interest and the PRAI are separated by the flexible eight amino acids long linker sequence GGAGGAGG (Figure 1, bottom panel). Whenever fusing a protein of interest to PRAI, it is recommended to test whether PRAI is still functional, even if this is very likely the case. This is straightforward, as documented in our study, as well as fast and low cost.

### 4.3. The RWDBD Remains Functional When Fused to PRAI

With a length of 224 amino acids, PRAI is relatively large, raising the possibility that its fusion to the C-terminus of a protein of interest may affect that protein’s function. Therefore, we next tested whether RWDBD can still execute its function when C-terminally decorated with PRAI. As indicated earlier, overexpressed RWDBD hampers Gcn2 activation. Gcn2 activity can be easily scored by monitoring growth on medium containing the drug 3-amino-2,4-triazole (3AT). 3AT inhibits the histidine (His) biosynthetic enzyme encoded by *HIS3*, leading to His starvation [21]. Cells able to activate Gcn2 are able to overcome starvation and grow on medium containing 3AT, while cells unable to activate Gcn2 cannot. Hence, growth on 3AT is indicative of the level of Gcn2 activation, where the growth rate is proportional to the level of Gcn2 activity.

To test whether the RWDBD still retains its function of inhibiting Gcn2 when fused to PRAI, yeast wild-type strain H1511 was transformed with plasmids expressing from the galactose-inducible promoter GST-RWDBD and GST-RWDBD^R2259A^, decorated with or without C-terminal PRAI-myc, respectively. As a control, H1511 and the isogenic *gcn1∆* strain (H2556) were transformed with a plasmid expressing GST only. A semiquantitative growth assay was conducted as above. On plates containing glucose, strains grew equally well. On plates containing galactose, strains expressing GST-RWDBD and GST-RWDBD^R2259^, decorated with or without C-terminal PRAI-myc, also grew as well as those expressing GST alone, suggesting that overexpression of the recombinant proteins did not affect cell growth in general (Figure 2B, left two panels). On medium containing galactose and 3AT, the *gcn1Δ* strain was barely able to grow, as expected, since the absence of Gcn1 does not allow Gcn2 activation (Figure 2B, row 9). Amino acid Arg-2259 in the RWDBD is critical for the interaction with Gcn2, rendering overexpressed RWDBD^R2259A^ unable to disrupt Gcn1-Gcn2 interaction and unable to hamper Gcn2 activation [12]. Accordingly, as expected, strains expressing GST-RWDBD^R2259A^ or GST-RWDBD^R2259A^-PRA1-myc were still able to grow on medium containing 3AT (Figure 2B, right two panels, row 6–8 vs. 1 and 2). In contrast, and as expected, strains expressing GST-RWDBD showed reduced growth compared to strains expressing GST (Figure 2B, right two panels, rows 4 and 5 vs. 1 and 2). The same growth defect was observed for the strain expressing GST-RWDBD-PRAI-myc (Figure 2B, right two panels, row 3 vs. 4 and 5). This strongly suggests that the RWDBD retained its function even when fused C-terminally with PRAI-myc. This also suggests that the PRAI-myc did not affect the proper folding of the protein, i.e., it retained its native solubility.

From the above findings, we can conclude that the RWDBD and PRAI both retained their function when fused in-frame. This would suggest that decorating the protein of interest with a C-terminal PRAI does not impact the function of either protein. Certainly, this needs to be validated for each protein of interest investigated. In addition to a flexible linker separating the protein of interest from the PRAI, the protein of interest is also separated from the N-terminal GST tag by a flexible linker, again with the intention to reduce the likelihood of steric hinderance that may interfere with the function of the protein of interest.

### 4.4. The Epitope Tags of the Protein Constructs Are Detectable

The Trp prototrophy conferred by PRAI fused C-terminally to the protein of interest would indicate that the protein of interest is expressed in full length. Western blotting could be conducted to verify the correct size of the hybrid protein by probing for the N- or C-terminal epitope tags. Particularly important are Western blots where variations in expression levels must be considered when assessing the functions of different protein variants. In our plasmid system, the recombinant proteins harbour an N-terminal GST tag and a C-terminal myc tag. Very strong antibodies against GST and myc are commercially available, and both tags also allow easy protein purification or co-precipitation studies. In fact, anti-myc antibody-linked resins are commercially available, and so are glutathione resins (GST binds to glutathione).

**Figure 3:**
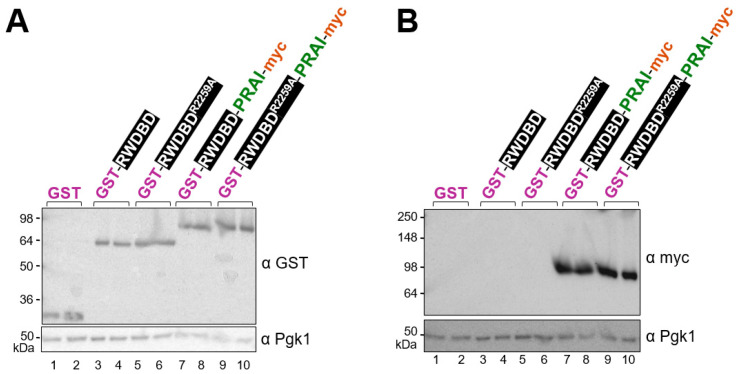
The GST and myc epitope tags are detectable in Western blots. Wild-type yeast H1511 was transformed with plasmids expressing from a galactose-inducible promoter the recombinant proteins as indicated (from left to right: plasmids pES128-9, pES124-B2, pES167-2E, pAG01, pSG50). Independent transformants were grown to the exponential phase in medium containing galactose as a carbon source and then harvested as outlined in the Materials and Methods Section. Whole cell extracts were generated, and equal volumes of extracts subjected to denaturing gel electrophoresis using two gradient gels with samples loaded in the same order. Immunoblotting was then performed using antibodies against (**A**) the GST tag and Pgk1 as loading control, or (**B**) the myc tag and Pgk1. Since the only goal was to determine the presence or absence of bands, densitometry measurements were not performed.

To detect the tags via immunoblotting, transformants were grown to exponential phase in liquid medium containing galactose as a carbon source. Cell extracts were generated and subjected to denaturing SDS polyacrylamide electrophoresis and Western blotting using antibodies against GST and myc, and the housekeeping gene Pgk1 as a loading control. When probing for GST, one distinct band could be detected in each lane, with those of GST alone and GST-RWDBD-PRAI-myc at the expected size of 26 and 95 kDa, respectively. For GST-RWDBD, the detected size was slightly higher than expected (69 kDa vs. 64 kDa) (Figure 3A). As reported previously, observed discrepancies between calculated and observed kDa values of a protein could be due to amino acid composition, post-translational modifications, protein conformation, and SDS binding, as well as variations in the accuracy of pre-stained protein markers and differences in gel composition or running conditions, which can affect protein mobility [22,23,24].

When probing for myc, expected signals were only observed in the cell extracts containing recombinant proteins harbouring a myc tag (Figure 3B). Together, this suggests that both tags are readily detectable via Western blotting.

While in Western blotting antibodies detect their epitope tag in denatured proteins, in immunoprecipitation or purification procedures, the tags need to be accessible within natively folded proteins. The N-terminal GST tag is commonly used for co-precipitation or purification approaches [25,26], and so is the myc tag [25,26]. To reduce the possibility of steric hindrance in our plasmid system, the tags are separated from the fusion protein by linkers, with the SGGGGG linker between the GST tag and the fusion protein, and the GGG linker between PRAI and the myc tag. Nevertheless, one cannot exclude the possibility that steric hindrance obstructs the accessibility of the tag in the context of a folded protein, hence it is recommended to experimentally test with each protein of interest whether the tags are accessible to antibodies or resins (e.g., glutathione-linked resins). At least for the GST-RWDBD hybrid protein, it has been shown previously that the GST tag can be used for co-precipitation studies [12]. Since the accessibility of tags needs to be tested for any protein of interest, we refrained from conducting co-precipitation studies and from testing other hybrid proteins in our study.

### 4.5. Enrichment of Yeast Cells Expressing Protein Variants with Desired Phenotypes

If, for example, the scenario was to identify protein variants with improved properties from a yeast library generated via random mutagenesis, the first step would be to remove cells that express truncated proteins. We have demonstrated above that our vector system allows the removal of yeast cells expressing truncated proteins due to their inability to grow in the absence of Trp.

The next desired step would be to enrich the library for those cells that express protein variants with improved properties. This can be conducted by competitive growth, as long as the improved protein property elicits a change in phenotype that provides a growth advantage. Such competitive growth conditions would need to be optimised first. This optimisation process is outlined next using as an example our proof-of-principle study.

In our study, the desired protein function is the loss of Gcn2 binding and concomitant gain of Gcn2 function, which manifests itself phenotypically by the strain’s regained ability to grow in the presence of 3AT. Two control strains were needed, the original control strain that expresses the original protein of interest, and the positive control strain that expresses a protein with the desired improved property. In our study, these were RWDBD and RWDBD^R2259A^, respectively. RWDBD can inhibit Gcn2, impairing growth on 3AT, while RWDBD^R2259A^ cannot. If a positive control protein is not available, a strain that displays an equivalent improved fitness can be used instead. In our scenario, this could have been a wild-type strain harbouring the vector alone and thus containing a fully active Gcn2, since we are looking for mutations that render RWDBD unable to bind Gcn2 and incapable of inhibiting Gcn2.

**Figure 4:**
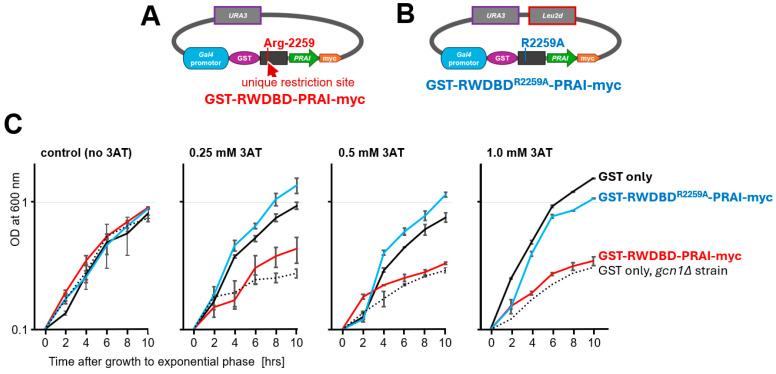
Determining growth conditions that provide a growth advantage for cells expressing RWDBD^R2259A^ over those expressing RWDBD. Wild-type yeast H1511 and isogenic strain H2556 were transformed with a plasmid expressing GST alone (plasmid pES128-9), and H1511 was transformed with one or the other plasmid expressing from a galactose-inducible promoter the recombinant proteins as indicated in (**A**) (plasmid pAG01) or (**B**), (plasmid pSG50), respectively. (**C**) Transformants were then grown overnight to exponential phase in medium containing galactose as a carbon source, re-inoculated in the morning into fresh medium containing 3AT at the indicated concentrations, and the OD measured at 600 nm at 2 h intervals. The average of two independent experiments with independent transformants is shown, and error bars are indicated.

RWDBD was expressed from a plasmid harbouring only the *URA3* selectable marker (pAG01, equivalent vector is pAG41 for the original control strain), while RWDBD^R2259A^ was expressed from a vector harbouring both selectable markers *URA3* and *Leu2d* (pSG50, equivalent vector is pAG40 for the positive control strain) (Figure 4A,B). These plasmids were introduced into the wild-type yeast strain H1511.

First, it was necessary to identify growth conditions under which the positive control strain harbouring RWDBD^R2259A^ grew at a faster rate than the original control strain harbouring RWDBD, i.e., had a higher fitness. The point of growth difference was the ability of a cell to activate Gcn2 under starvation conditions, which is hampered by RWDBD but not RWDBD^R2259A^. Therefore, we determined the optimal 3AT concentration at which there was a clear difference in growth rate between these two control strains. To do this, saturated overnight cultures of the strains were individually inoculated into medium containing galactose and increasing amounts of 3AT as before, the strains grown at 30 °C and 180 rpm overnight, the exponentially growing cultures re-inoculated into fresh medium containing the same amount of 3AT as before, and the optical density measured at 2 h intervals. As a control, we also included in our growth assays the wild-type strain and isogenic *gcn1Δ* strain expressing GST alone.

As expected, in the absence of 3AT, all strains grew at a similar rate (Figure 4C). However, the *gcn1Δ* strain did not grow as well as the wild-type strain expressing GST alone in the presence of 0.25 mM 3AT (Figure 4C). The *gcn1Δ* strain still showed some growth, likely because the amount of 3AT used was insufficient to fully inhibit His biosynthesis. The strain expressing GST-RWDBD^R2259A^ grew at least as well as the wild-type strain expressing GST alone, in agreement with the idea that RWDBD^R2259A^ is unable to impair Gcn2 activation, allowing the cell to overcome starvation and grow in the presence of 3AT. In agreement with previous findings [12], the strain overexpressing RWDBD showed impaired growth in the presence of 3AT, as found for the *gcn1Δ* strain, due to RWDBD impairing Gcn2 activation. The growth discrepancy between strains expressing RWDBD and RWDBD^R2259A^ became slightly more pronounced with increasing 3AT concentrations. For the competition study, we chose the lowest 3AT concentration that still elicited the maximum difference in growth, which was 0.5 mM.

The next step would be to perform a pilot competitive growth assay to determine the timeframe over which the culture becomes enriched with cells displaying the desired phenotype (the positive control strain), i.e., expressing the protein with the desired improved property. Galactose medium containing 0.5 mM 3AT was inoculated with cells to a final OD_600_ of 0.05. The inoculum consisted of a 1:9 ratio, with 1 part positive control strain (cells expressing RWDBD^R2259A^) and 9 parts original control strains (cells harbouring RWDBD). For the control growth assay, the same experimental setup was used, but the medium did not contain any 3AT. After every 22–24 h of growth, an aliquot was taken and the culture re-inoculated at an OD_600_ of 0.05 into fresh galactose medium containing 0.5 mM 3AT (or not containing 3AT in case of the control growth assay). The aliquots were 10-fold serially diluted and spread on solid media lacking Ura or lacking both Ura and Leu. Plates were incubated at 30 °C until colonies emerged, and the number of colonies determined. All strains were expected to grow on plates lacking Ura, while on plates lacking Ura and Leu, only strains that harboured the plasmid bearing RWDBD^R2259A^ (the positive control strain) were expected to grow since its backbone carries the *Ura3* and *Leu2d* selectable markers. This allowed us to determine in the competitive growth culture the ratio of cells expressing GST-RWDBD^R2259A^ (positive control strain) to those expressing GST-RWDBD (original control strain), and to follow over time any changes from the initial 1:9 ratio.

**Figure 5:**
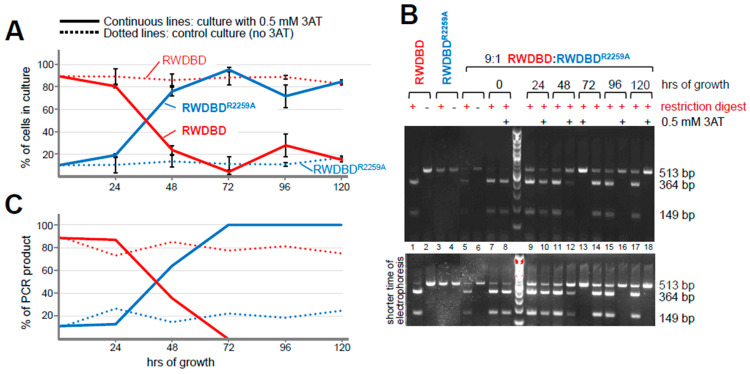
Competitive growth assay demonstrating the successful enrichment of cells expressing the protein of interest with enhanced features. A mixed culture was generated in medium containing 0.5 mM 3AT and galactose as a carbon source, with one part of wild-type yeast H1511 expressing RWDBD^R2259A^ and nine parts of the same strain but expressing RWDBD (plasmids pSG50 and pAG01, respectively). An identical culture was inoculated in medium containing galactose but not 3AT. The cultures were grown at 30 °C and 180 rpm for 22–24 h, and then re-inoculated into the same medium, again with a starting OD_600_ of 0.05. After 22–24 h, the re-inoculation was repeated as before, until 120 h (5 days) had passed. Before re-inoculating the culture into fresh medium, (**A**) an aliquot of the culture was taken, serially diluted, and plated on medium containing Leu and Ura or only Ura to determine the colony forming units. All strains grew on plates containing Ura and Leu, while on medium containing only Ura, only cells that harboured the RWDBD^R2259A^-expressing plasmid grew (see Figure 4A,B). From this, it was possible to deduce the cell numbers in the competitive growth culture and, consequently, the percentage abundance of cells expressing RWDBD or RWDBD^R2259A^. The average % abundance from two independent experiments using independent transformants is displayed as a graph, and the standard errors are indicated. (**B**) A 1 µL volume of each culture aliquot was used in PCR using primers ES2964r and ES2963s. Then, an aliquot of the PCR solution was subjected to an overnight *Ase*I restriction digest at 37 °C and resolved via agarose gel electrophoresis. (**C**) The intensity of the bands in (**B**) was measured using ImageJ software version 1.54 m [27]. Then, the ratio of intensity of the 513 bp band (representing RWDBD^R2259A^) versus the two 364 and 149 bp bands (representing the digested RWDBD amplicon) was determined and the abundance of the amplicons plotted in a graph as percentage.

According to the colony counting results, as expected, the strain expressing RWDBD dominated at 90% at the start (Figure 5A). After 72 h, its abundance reduced to 4%; accordingly, the strain expressing RWDBD^R2259A^ became the prominent strain (Figure 5A). It appears that the point of equal ratio between the two strains was between 24 and 48 h. Since the strain expressing RWDBD remained the predominant strain on medium lacking 3AT over the equivalent time period (89% abundance), and since after 120 h this was still the case (83% abundance), this supports the idea that the selection pressure imposed by 3AT drove the change in the ratio between cells expressing RWDBD vs. RWDBD^R2259A^. Taken together, these results suggest that 72 h of competitive growth were sufficient to dramatically enrich the strain with the desired phenotype.

Next, we wanted to obtain additional evidence of the effectiveness of the competitive growth procedure in enriching the strain with the desired phenotype. The idea behind the next assay is that the more abundant a strain is in the competitive growth culture, the more abundant its plasmid should be; hence, PCR applied directly on an aliquot of the competitive growth culture should yield a larger proportion of amplicons originating from the more abundant plasmid. To test this, we used PCR primers that amplified a portion of the RWDBD that encompasses the amino acid Arg-2259, the point of difference between the RWDBD and the RWDBD^R2259A^ proteins. Another difference was that the R2259A substitution removed the nearby *AseI* restriction site. This meant that the PCR amplicons originating from the RWDBD versus the RWDBD^R2259A^ templates could be distinguished by a subsequent *AseI* restriction digest, where only the amplicons from the RWDBD template were cut but not the amplicons from the RWDBD^R2259A^ template; hence, after resolving the digested amplicons via agarose gel electrophoresis, they could easily be identified and quantified (Figure 5B, lanes 1 and 2 vs. 3 and 4). The same was true for a mixed sample where the RWDBD^R2259A^ and RWDBD templates were in a 1:9 ratio (Figure 5B, lanes 5 vs. 6). At the start of the competitive growth assay, restriction digest of the PCR amplicons revealed that the 364 and 149 bp bands (PCR product containing the restriction site) were more abundant than the 513 bp band (PCR product lacking the restriction site) (Figure 5B, lanes 7 and 8), suggesting that the strains expressing RWDBD were more abundant, as expected. Upon quantification of the band intensities, the RWDBD abundance was 90% and 88.5% for the competitive growth and control culture, respectively, which aligned well with the expected 90% (Figure 5C). After 48 and 72 h of growth, the 513 bp PCR product originating from the RWDBD^R2259A^ template increased in abundance compared to the bands originating from the RWDBD template (Figure 5B, lanes 10 vs. 12 vs. 13). Quantitation of the bands revealed that the strains expressing RWDBD^R2259A^ increased to 64% and then to almost 100% abundance. Growth beyond 72 h resulted in the positive control strain maintaining a level of 100%. Together, this indicated that 72 h of competitive growth was sufficient to enrich for the positive control strain expressing RWDBDR^2259A^.

In the control culture lacking 3AT in the medium, the 364 and 149 bp bands (PCR product originating from RWDBD) remained more abundant than the 513 bp band (PCR product originating from RWDBD^R2259A^) over time (Figure 5B, lanes 7, 9, 11, 14, 15, 17). The starting 10:90% ratio between the positive and original control strains only changed to a ratio of about 25:75% and 17:83% after 72 and 129 h, respectively, as opposed to 0:100% after just 72 h in the competitive growth culture grown in the presence of 3AT (Figure 5C). This means that the positive control strain was barely enriched under conditions lacking 3AT, supporting the idea that the enrichment of the positive control strain was not by chance but due to the selection pressure imposed by 3AT. This is in agreement with the colony counting results above (Figure 5A).

In summary, the colony counting approach and the liquid culture PCR-based assay gave similar results. Both assays indicated that the 10:90% ratio between the positive and original control strains shifted to a 50:50% ratio between 24 and 48 h, and that at 72 h, the abundance of the positive control strain was almost 100%. This suggests that a competitive growth of only a few days (here 3 days) was sufficient to enrich for strains with higher fitness, expressing the desired protein of interest with improved properties. Beyond 72 h, the colony-counting-based assay seemed to indicate that the ratio shifted from 4:96% ± 2% back to about 20:80% (28:72% ± 10.21% at 96 h and 15:85% ± 1.6% at 120 h), suggesting that competitive growth after reaching the point of maximal positive control strain proportion (here at 72 h) may not be advantageous. Nevertheless, the desired strain still remained the majority. On the other hand, the PCR-based assay seems to indicate that the 0:100% ratio remained unchanged beyond the 72 h of competition. However, it is possible that ethidium bromide-based staining may make it hard to detect low-abundant DNA in the gel. Nevertheless, the PCR-based assay still allows for the determination of whether the positive control strain became enriched in the culture. Another important advantage of the PCR-based assay is that—unlike a colony-based assay—it provides results in less than 24 h. This is critical for determining whether to continue a competitive growth culture, as it needs to be re-inoculated every 24 h. In contrast, a colony-based assay takes up to 2 days, making it too slow for timely analysis during an ongoing competitive growth assay. However, to use a PCR-based assay, a DNA sequence that is unique to the original strain or to the strain with improved property is essential, e.g., by way of presence or absence of a restriction site.

Following the competition assay, one could then identify the gene mutations responsible for providing increased strain fitness by performing targeted sequencing using next-generation sequencing techniques. The technique used can be amplicon sequencing, third-generation, or massive parallel sequencing, depending on the length of the DNA to be sequenced [28,29,30,31]. If there are several protein variants that enhance fitness, next-generation sequencing will not only reveal the sequence of the gene of interest variants eliciting enhanced fitness, but also reveal the relative abundance of the respective plasmids in the culture. Strains containing the plasmid-encoded protein conferring the highest fitness would be the most abundant.

## 5. Conclusions

We have developed a vector system that allows fast, low-cost, and non-laborious removal of cells expressing truncated versions of a protein of interest that may have emerged during random mutagenesis. With the open reading frame of the protein of interest being in-frame with the open reading frame of PRAI, the expressed fusion protein confers Trp prototrophy. Introduction of a stop codon or a frameshift in the open reading frame of the gene of interest would prevent translation of the downstream sequence coding for PRAI, thereby rendering the host cell Trp auxotrophic. Hence, on medium lacking Trp, the host cell is unable to grow and multiply, allowing easy selection against cells expressing these unwanted proteins. Mutations could not only introduce stop codons in an open reading frame but also lead to an amino acid substitution that renders the protein unstable and highly prone to degradation. Cells expressing such proteins can be selected against as well, given that the low abundance of the hybrid protein may be insufficient to confer full Trp prototrophy.

The two developed plasmids differ in the presence or absence of a second selectable marker, which enables the optimisation of a competitive growth assay using a colony-counting-based method. We have also demonstrated that a PCR-based method can be used to track changes in the relative proportions of cells expressing different proteins during competitive growth.

Our plasmid system was primarily established to remove cells expressing unwanted proteins of interest, i.e., those that are truncated, have a shift in their reading frame, or are hardly expressed. However, our system offers additional opportunities for applications. For example, the protein of interest—fused C-terminally to PRAI—is also equipped with epitope tags, allowing easy Western blotting, interaction studies, and protein purification. Flexible linker regions separating the protein of interest from the N-terminal GST tag and the C-terminal PRAI-myc portion reduce the possibility of affecting protein folding or protein function. Though unlikely, it is necessary to ascertain that the function of every protein of interest is not impaired when fused to GST and PRAI-myc. For protein function studies, the GST tag can be removed via the existing protease site thrombin, if necessary. Another protease site could be introduced immediately downstream of the sequence coding for the protein of interest to remove the C-terminal PRAI-myc portion. Further variations of our constructs are possible to adapt to specific research questions. For example, if desired, this system could be integrated into the chromosome rather than maintained on a plasmid. Furthermore, the galactose-inducible promoter could be replaced by a different one, and the tags could be removed or replaced by other tags. The *TRP1* gene could be replaced by one conferring drug resistance (e.g., G418 resistance) instead of prototrophy.

Some research questions may require generating a series of truncated proteins of interest, rather than full-length mutated proteins; for example, mapping the location of a specific function within a protein. In this scenario, after generating a random mutagenesis or truncation library, it would be desirable to enrich for C-terminally truncated protein variants rather than full length ones. Mutations leading to a stop codon or a frameshift would prevent translation of the C-terminal extension encoding PRAI, which could be used to select for Trp auxotrophic cells. In contrast to Trp prototrophic cells, Trp auxotrophic strains are resistant to the drug 5-fluoroanthranilic acid (5-FAA) [32]. 5-FAA is an analog of anthranilic acid, a precursor in the tryptophan biosynthesis pathway that exerts its toxicity through its conversion into the antimetabolite 5-fluorotryptophan. Accordingly, strains expressing full-length proteins of interest fused to PRAI would be sensitive to 5-FAA, while strains expressing truncated versions of the protein of interest (lacking the C-terminally attached PRAI) would be resistant to this drug. To validate that a strain is stably expressing a truncated variant of the protein of interest, rather than no protein variant at all, one could probe for the remaining N-terminal GST tag, for example, using a fast colony Western assay [33].

## Data Availability

The original contributions presented in this study are included in the article/Supplementary Material. Further inquiries can be directed to the corresponding author.

## Supplementary Materials

The following supporting information can be downloaded at: https://www.mdpi.com/article/10.3390/biom15030412/s1, Figure S1: Original images of Figure 3, Figure S2: Original images of Figure 5B,C, as well as plasmid maps for pAG40 and pAG41 can be found in supplementary materials.

## Abbreviations

3AT: 3-amino-1,2,4-triazole; Gcn: General control non-derepressible; PRAI: Phosphoribosylanthranilate isomerase; RWD: a domain found in RING finger-containing proteins, WD-repeat-containing proteins, and yeast DEAD (DEXD)-like helicases; RWDBD: RWD-binding domain.
