# Supplementary material for: A Plasmid System That Utilises Phosphoribosylanthranilate Isomerase to Select Against Cells Expressing Truncated Proteins
Source: Biomolecules. 2025 Mar 14;15(3):412. doi: 10.3390/biom15030412 (PMC11940383; doi:10.3390/biom15030412)
Supplement: Supplementary file 1 [file biomolecules-15-00412-s001.zip › Ghuge-TRP1-FIGURE ESi-final-rawData.pdf]

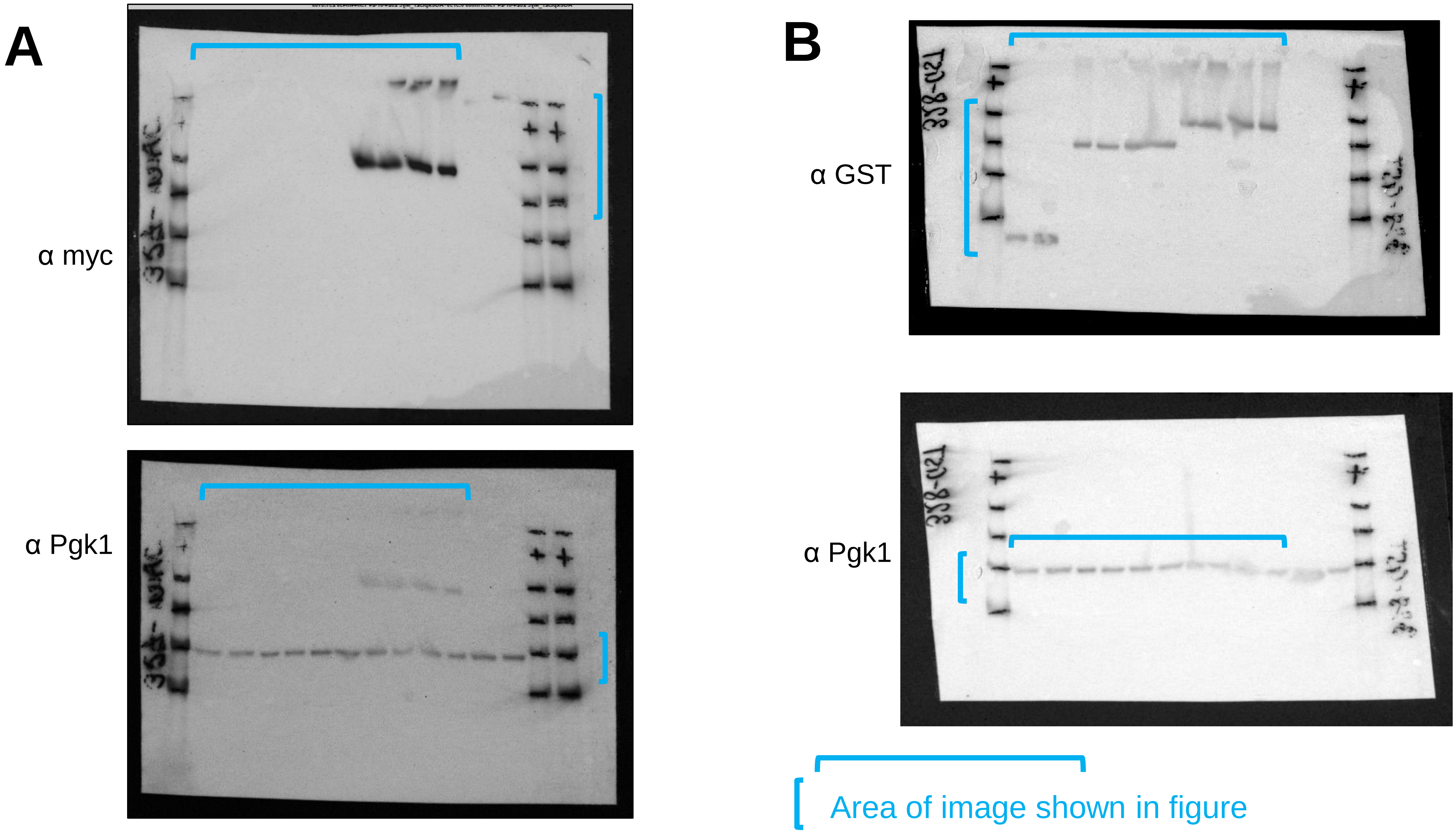

Figure S1. Figure 3 – raw data

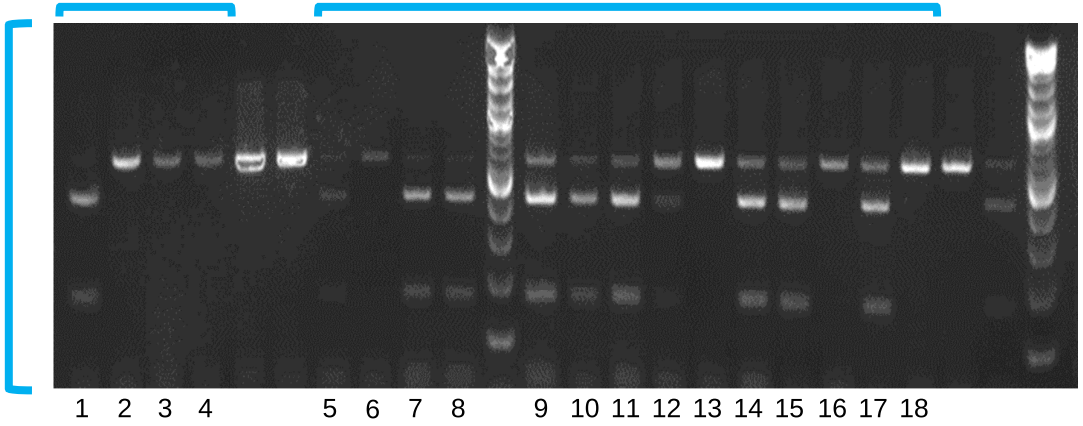

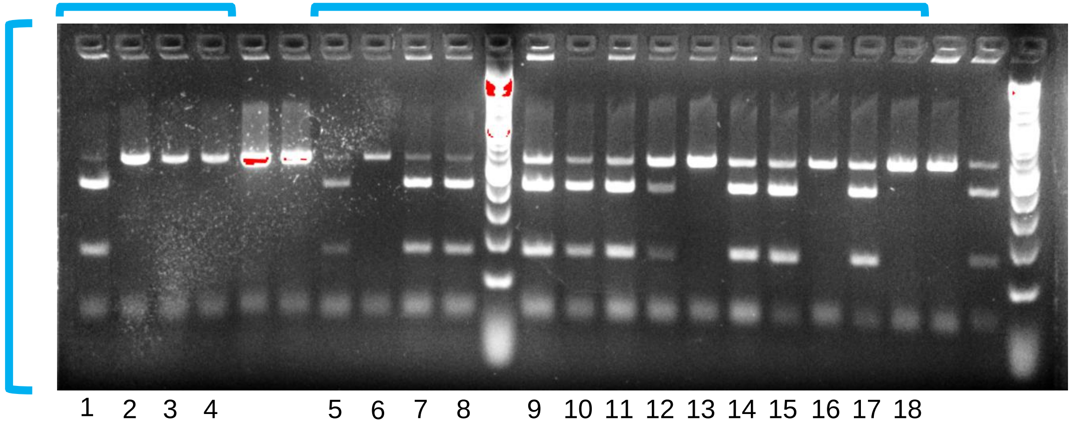

Area of image shown in figure

lane #	hrs of growth	AseI digest	0.5 mM 3AT	quantitation of band intensity 530bp	365bp	125	365+125	total	determine % abundance RWDBD^{R2259A} 365 bp	RWDBD 365+125 bp	all bands
7	0	yes	no	2416.4	16059.6	5764.9	21824.4	24240.9	10.0	90.0	100.0
8		yes	yes	2957.0	17170.7	5653.3	22824.0	25781.0	11.5	88.5	100.0
9	24	yes	no	8587.4	17037.7	6462.8	23500.5	32087.9	26.8	73.2	100.0
10		yes	yes	2025.0	10038.4	3432.3	13470.8	15495.8	13.1	86.9	100.0
11	48	yes	no	3287.4	13089.4	5619.5	18708.9	21996.3	14.9	85.1	100.0
12		yes	yes	6337.0	2349.9	1201.3	3551.2	9888.2	64.1	35.9	100.0
13	72	yes	yes	10409.5	0.0	0.0	0.0	10409.5	100.0	0.0	100.0
14		yes	no	5053.1	12484.4	4899.7	17384.1	22437.2	22.5	77.5	100.0
15	96	yes	no	4020.1	13045.1	4457.8	17502.9	21523.0	18.7	81.3	100.0
16		yes	yes	6489.5	0.0	0.0	0.0	6489.5	100.0	0.0	100.0
17	120	yes	no	5829.5	12498.8	5050.5	17549.3	23378.7	24.9	75.1	100.0
18		yes	yes	11353.7	0.0	0.0	0.0	11353.7	100.0	0.0	100.0

Figure S2. Figure 5B,C – raw data
